# Effectiveness of Chickpeas on Blood Sugar: A Systematic Review and Meta-Analysis of Randomized Controlled Trials

**DOI:** 10.3390/nu15214556

**Published:** 2023-10-27

**Authors:** Taegwang Nam, Anna Kim, Yongtaek Oh

**Affiliations:** 1 College of Korean Medicine, Woosuk University, Jeonju 54986, Republic of Korea; skaxorhkd12@naver.com; 2 KM Data Division, Korea Institute of Oriental Medicine, Daejeon 34054, Republic of Korea

**Keywords:** chickpea, cicer arietinum, diabetes, blood sugar, insulin

## Abstract

Diabetes affects one in eleven adults globally, with rising cases in the past 30 years. Type 1 and type 2 cause blood sugar problems, increasing cardiovascular risks. Dietary control, including chickpeas, is suggested but needs more research. Comprehensive searches were conducted across multiple databases for the randomized controlled trial efficacy of chickpea consumption to lower blood sugar levels to a healthy range, with data extraction and risk of bias assessment performed independently by two researchers. Statistical analysis was performed using RevMan 5.4, expressing continuous data as mean differences and risk ratios with 95% confidence intervals, and a summary of the findings is provided considering the variations in study characteristics. A total of 118 articles were initially identified from seven databases, primarily from Anglo–American countries, resulting in 12 selected studies after the identification and screening processes. These studies involved 182 participants, focusing on healthy or normoglycemic adults, and assessed the effects of chickpeas compared to various foods such as wheat, potatoes, pasta, sauce, cheese, rice, and corn. A meta-analysis involving a subset of studies demonstrated that chickpeas were more effective in reducing blood glucose iAUC compared to potatoes and wheat. Chickpeas offer the potential for blood sugar control through low starch digestibility, high fiber, protein, and hormonal effects. Although insulin benefits are seen, statistical significance varies, supporting their role in diabetic diets focusing on nutrient-rich foods over processed carbs.

## 1. Introduction

Approximately one in eleven adults worldwide have diabetes, making it four times more prevalent over the past 30 years, and it ranks as the ninth leading cause of death [1]. Diabetes is a disease that involves disorders in the endocrine metabolism responsible for blood sugar regulation and can be broadly categorized into type 1 and type 2 diabetes. Type 1 diabetes occurs due to the autoimmune destruction of beta cells in the pancreas, which is responsible for insulin production, and it is influenced by genetic factors and yet-to-be-determined environmental factors [2]. On the other hand, type 2 diabetes is a condition characterized by insulin resistance in peripheral organs, caused by factors such as obesity, unhealthy diet, sedentary lifestyle, smoking, stress, and depression [1,3].

Both type 1 and type 2 diabetes have different causes, but the common characteristic is the inability to regulate blood sugar within the normal range. Maintaining high blood sugar levels leads to various health problems. In particular, hyperglycemia has been found to contribute to numerous cardiovascular diseases, such as myocardial infarction, stroke, and dementia, and it is associated with visual impairment and cancer [4,5,6,7,8].

Regarding diabetes, the American Diabetes Association’s Standard of Care recommends dietary management [9]. Dietary management in diabetes is cost-effective and can increase insulin response without raising plasma glucose levels [9]. It also improves cardiac metabolism and helps achieve energy balance [9]. In particular, research has shown that the glycemic index (GI) and glycemic load on fasting glucose levels are related, and consuming foods with a low GI, such as varieties of legumes and dairy products, has been found to reduce glycemic load and have positive effects on cardiovascular diseases and body weight [10,11,12,13].

Functional foods that utilize dietary adjustments and various factors, such as specific nutrient regulation, edible oils, mushrooms, herbs, and vitamins, have been widely used for treating diabetes and improving present treatment outcomes [2,14,15,16,17,18,19,20,21,22].

Many studies have shown that pulse crops, including chickpeas and processed food products derived from them, have a low GI [23,24,25,26]. Furthermore, numerous studies have demonstrated that pulse crops, including chickpeas, aid in blood sugar regulation [27,28,29]. These research findings imply that incorporating pulse crops, including chickpeas, into diabetes management diets can improve the health status of diabetic patients.

Chickpeas are rich in protein, with a high proportion of essential and non-essential amino acids. Notably, their protein bioavailability surpasses that of other legumes. The unique chemical composition of chickpeas, coupled with their low glycemic index, is presumed to have a positive impact on blood sugar by reducing carbohydrate bioavailability and absorption rates.

Indeed, based on these distinctive features, chickpeas are often referred to as a superfood, and global chickpea consumption has been experiencing explosive growth. In the United States, annual chickpea consumption more than doubled from 1.9% in 2003 to 4.5% in 2018 [30]. Furthermore, the global chickpea market is expected to increase from USD 9.15 billion, currently, to USD 10.68 billion by 2028 [31].

However, there is currently a lack of detailed studies on the specific efficacy of chickpeas in relation to diabetes. Most systematic reviews and meta-analyses have only focused on studies related to the pulse crop group, and there is a shortage of studies on chickpeas. Similarly, there has been a lack of research on the specific effects of chickpeas alone on diabetes [28,32,33]. Considering these aspects, we have determined the need to clearly elucidate the impact of chickpeas on diabetes and blood sugar. Therefore, through this study, we aim to provide clear insights into the influence of chickpeas on blood sugar regulation.

## 2. Materials and Methods

### 2.1. Protocol and Registration

Our systematic review protocol was registered in the international prospective register of systematic reviews under the registration number PROSPERO (CRD42023428211).

### 2.2. Data Sources and Searches

We comprehensively searched the following eight English and Korean electronic databases: Cochrane Central Register of Controlled Tests (via Cochrane library), Medline (via PubMed), EMBASE (via Elsevier), KoreaMed, Riss, Kiss, ScienceON, and OASIS up to April 2023.

All studies retrieved from various databases and identified through additional sources were imported into a Microsoft Office Excel spreadsheet. Subsequently, duplicate entries were systematically eliminated. Following this, the titles and abstracts of the articles were meticulously examined for initial inclusion criteria. For studies meeting these criteria, their full-text versions were obtained and subjected to a thorough review to make the final inclusion decision. The search strategy and results for each database are presented in Table A1, Table A2, Table A3, Table A4, Table A5, Table A6 and Table A7.

### 2.3. Study Selection

#### 2.3.1. Study Design

Only crossover group randomized controlled trials (RCTs) assessing the efficacy of chickpeas (scientific name: *Cicer arintium*) for high blood sugar were included. We excluded non-randomized trials and crossover studies to reduce the risk of potential bias. There were no limitations regarding the publication language of the studies.

#### 2.3.2. Participants

Studies involving type 2 diabetic patients were included. Studies involving healthy people were also included. Studies in which the ingredients used in the diet were not clear or chickpeas were not used alone were excluded. There were no limitations regarding the sex, race, and nationality of the participants.

#### 2.3.3. Interventions

For treatment interventions, studies involving chickpea-processed meals, such as chickpea-based diet, chickpea flour bread, macaroni and chickpeas, pasta and sauce with chickpeas, chickpeas, chickpeas with rice, 60% cellular chickpea powder, and chickpea-extruded snacks, for the treatment of high blood sugar were included. However, studies that did not use a single ingredient of chickpeas were excluded. For control interventions, studies with placebo, wheat-based diet, white bread, macaroni and cheese, pasta and sauce, plain white rice, small potato portion, mashed potato, 0% cellular chickpea powder, and corn were included.

#### 2.3.4. Outcome Measures

In this study, we considered plasma glucose as a primary outcome. Secondary outcomes included plasma insulin.

### 2.4. Data Extraction

In the case of the studies ultimately included, we employed a standardized Excel form that had been pilot-tested to extract the following key information: essential details, such as the first author’s name, their country of origin, and the publication year; the sample size; participant characteristics; descriptions of the treatment and control interventions; specifics regarding the employed outcome measures and statistical analyses; outcomes; and data necessary for evaluating the risk of bias.

The entire process of study selection and data extraction was carried out independently by two researchers. In instances where the available data were unclear or insufficient, we made efforts to contact the authors of the included studies via email, if feasible, in order to obtain the necessary clarifications or additional information.

### 2.5. Risk of Bias Assessment

Two independent researchers evaluated the ROB for the included RCTs based on the Cochrane Collaboration’s ROB tool. The Cochrane Collaboration’s tool comprises seven domains; however, we assessed the following six domains: (1) random sequence generation, (2) allocation concealment, (3) blinding of participants, (4) blinding of assessors, (5) incomplete outcome data, and (6) selective outcome reporting. Domains (1) and (2) assess for selection bias level. Domain (3) assesses for performance bias, and (4) for detection bias. Domain (5) assesses for attrition bias, and (6) for reporting bias. For each domain, the ROB was rated as low risk (L), high risk (H), or unclear (U). 

### 2.6. Data Analysis and Synthesis

Statistical analysis was performed using RevMan 5.4 (version 5.4 for Windows; the Nordic Cochrane Center, Copenhagen, Denmark). Continuous data were presented as mean differences and risk ratios, accompanied by their respective 95% confidence intervals. For robustness, a sensitivity analysis was pre-planned to assess the potential influence of methodological quality, particularly focusing on trials with a low risk of bias (ROB). In cases where substantial variation in study characteristics prevented a meta-analysis, we provided a comprehensive summary of the findings in the results section to elucidate the study outcomes and their implications.

## 3. Results

### 3.1. Study Selection and Description

A total of 118 articles were identified from seven electronic databases (English databases: *n* = 79, Korean databases, *n* = 39).

Almost all studies were conducted in Anglo–American countries. Five studies were conducted in Canada, two studies were conducted in the UK, and two studies were conducted in Australia. One study was conducted in the USA.

**Figure 1:**
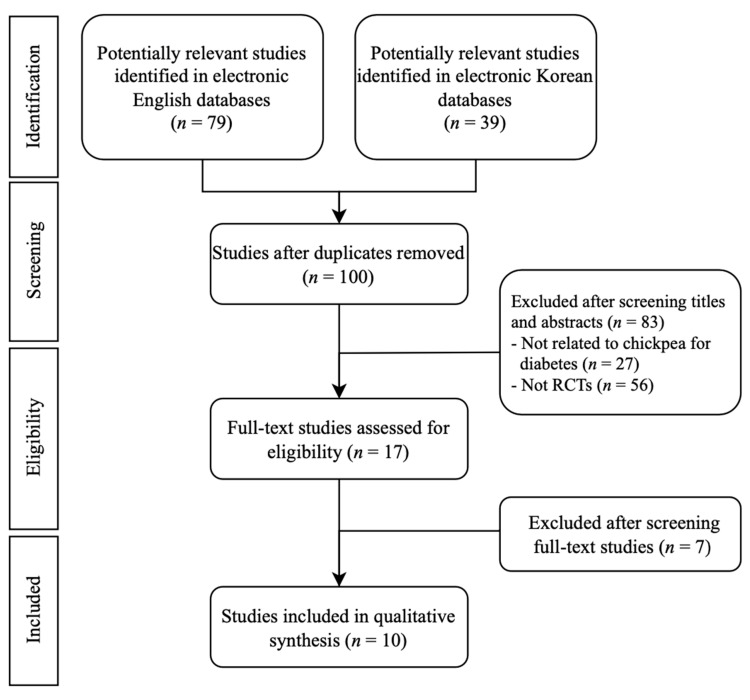
Flowchart of the RCT selection process. RCTs: randomized controlled trials.

After going through the identification, screening, and resilience processes, ten papers were finally selected. Table 1 summarizes the nutritional ingredients of control groups and intervention groups. Table 2 summarizes the details of the included studies. Figure 1 shows a flow chart of the study selection process as recommended in the Preferred Reporting Items for Systematic Reviews and Meta-Analyses guidelines. Meta-analysis of six RCTs was performed, focusing on groups that performed the same intervention. One RCT was excluded because the outcome assessment was different [34]. Three RCTs were excluded because the control interventions were not unified [35,36,37].

### 3.2. Participants

A total of 182 participants were included. All participants were healthy adults or normoglycemic adults. Most studies recruited both men and women, but some studies recruited participants for each single gender.

### 3.3. Outcomes

The included studies reported various outcome measures. Glucose iAUC (blood glucose iAUC, plasma glucose iAUC) and insulin iAUC were recorded in all studies.

#### 3.3.1. Chickpeas versus Wheat

Four RCTs compared chickpeas with wheat [34,35,37,43]. Four RCTs showed that the blood glucose iAUC or plasma glucose iAUC of chickpeas were significantly lower than that of wheat. In fact, in the case of blood sugar iAUC, the chickpea group was 47.01%, 20.15%, and 47.14% lower than the wheat group, respectively, and in the case of 1 h HOMA-IR after meals, the chickpea group was 25.8% lower than the wheat group [34,35,37,43]. Two RCTs showed that the insulin iAUC of chickpeas was not significant. One RCT showed that the insulin iAUC of chickpeas was significantly higher than that of wheat (12.32%) [37], and another RCT showed that the insulin iAUC of chickpeas was significantly lower than that of wheat (43.84%) [43].

#### 3.3.2. Chickpeas versus Potatoes

Two RCTs compared chickpeas with mashed potatoes [39,40]. Two RCTs showed that the blood glucose iAUC of chickpeas was significantly lower than that of potatoes. In fact, in the study results, the chickpea group had 63.45% and 75.23% lower blood sugar iAUC than the potato group, respectively [36,42].

#### 3.3.3. Chickpeas versus Pasta

Two RCTs compared chickpeas with pasta [34,38]. It showed that the blood glucose iAUC of chickpeas was significantly lower than that of pasta and sauce. In fact, in the study results, the chickpea group had 35.45% and 78.25% lower blood sugar iAUC than the pasta group, respectively [38,40].

#### 3.3.4. Chickpeas versus Cheese

One RCT compared chickpeas with cheese [39]. It showed that the blood glucose iAUC of macaroni and chickpeas was significantly lower than that of macaroni and cheese. In fact, in the study results, the chickpea group had 22.17% lower blood sugar iAUC than the cheese group [39].

#### 3.3.5. Chickpeas versus Rice

One RCT compared rice and chickpeas with rice only [36]. It showed that the blood glucose iAUC of chickpeas with rice was significantly lower than that of rice only. However, there is no significant difference in insulin iAUC between chickpeas with rice and rice only. In fact, in the study results, the chickpea group had 41.84% lower blood sugar iAUC than the rice group [36].

#### 3.3.6. Chickpeas versus Corn

One RCT compared chickpeas with corn [41]. It showed that the plasma glucose iAUC and insulin iAUC of chickpeas were significantly lower than that of corn. In fact, in the study results, the chickpea group had 11.06% lower blood sugar iAUC than the corn group [41].

### 3.4. Assessment for Risk of Bias

**Figure 2:**
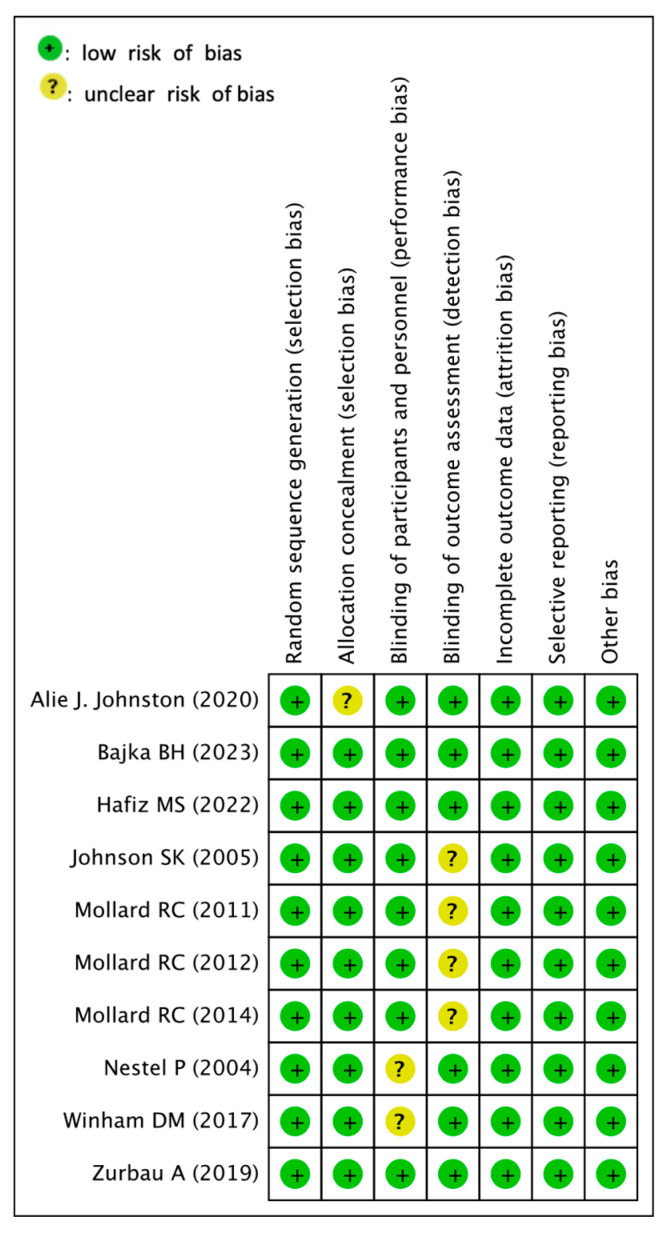
Risk of bias assessment [34,35,36,37,38,39,40,41,42,43].

Figure 2 summarizes the details of the risk of bias (ROB) for each RCT. Regarding the randomization procedure, the random sequence generation biases of all included studies were low. Due to the nature of the interventions, allocation conception biases in one study [41] and blinding of partner biases in two included studies [34,36] were found to be unclear. In other studies, homogenization was performed, and allocation biases and performance biases were low. The blinding outcome assessment bias between the four studies was unclear [35,37,38,39]. Other studies have gone through the process of double blind, with low attrition biases. In all studies, incomplete outcome data and selective reporting biases were low.

### 3.5. Meta-Analysis or Quantitative Analysis of the Included Articles

**Figure 3:**
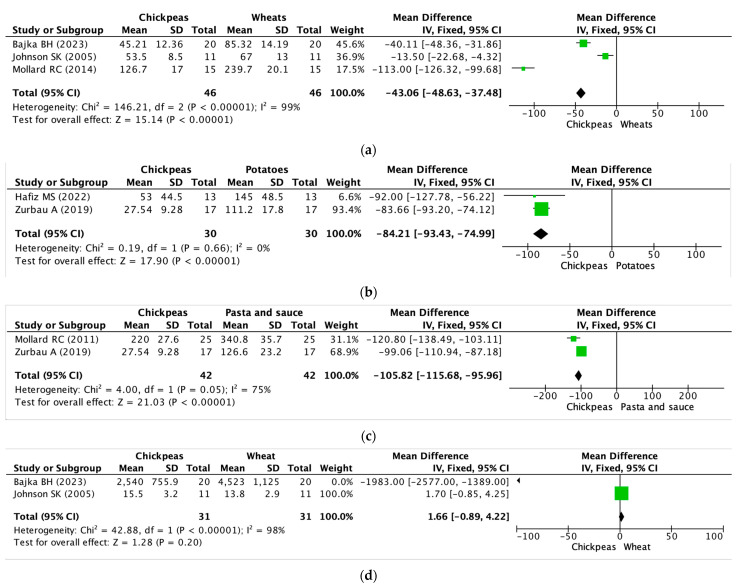
Effects of chickpeas on blood glucose iAUC and insulin iAUC. (**a**) Blood glucose iAUC of chickpeas and wheat groups. (**b**) Blood glucose iAUC of chickpeas and potatoes groups. (**c**) Blood glucose iAUC of chickpeas and pasta groups. (**d**) Insulin iAUC of chickpeas and wheat groups. iAUC: integrated area under the curve. Block: mean difference of the primary study, Diamond: pooled estimated mean of effect sizes obtained through meta-analysis [35,37,38,40,42,43].

A meta-analysis was conducted using a total of six studies that met the inclusion criteria. As a result, the mean difference (MD) was calculated (%). Figure 3 summarizes the details of the meta-analysis for six studies. The meta-analysis of three trials involving 46 participants in total showed that chickpeas were more effective in reducing blood glucose iAUC compared to wheat (a) (MD: −43.06, 95% CI: −48.63 to −37.48, *I*^2^: 99%). However, caution is advised when interpreting the difference in blood glucose iAUC between chickpeas and wheat, as the heterogeneity exceeds 75%. While these results are valid, it is important to note that the meta-analysis graphs do not contain 0 and are skewed towards the experimental group. Similarly, the meta-analysis of two trials involving 30 participants in total demonstrated that chickpeas were more effective in reducing blood glucose iAUC compared to potatoes (b) (MD: −84.21 95% CI: −93.43 to −74.99, *I*^2^: 0%). Also, meta-analysis of two trials involving 42 participants in total showed that chickpeas were more effective in reducing blood glucose iAUC compared to pasta groups (c) (MD: −105.82, 95% CI: −115.68 to −95.96, *I*^2^: 75%). An additional meta-analysis was carried out, covering two trials that included a total of 23 participants. Nevertheless, there were no statistically significant findings regarding differences in insulin iAUC between consuming chickpeas and wheat.

Furthermore, there was a notable level of inconsistency observed during the two trials, indicated by an *I*^2^ value of 98% and exacerbated by the inclusion of a value of 0 in the meta-analysis graph.

## 4. Discussion

According to the research findings, chickpeas have been shown to have a positive impact on blood sugar management compared to other common foods. Additionally, while not statistically significant in the meta-analysis results, some studies have indicated a positive effect of chickpeas on insulin.

In fact, the low digestibility and high resistance of starch in legumes, along with high levels of amylose and dietary fiber, contribute to delivering less glucose to the circulatory system, thus aiding in lowering blood sugar levels [44]. Particularly, the characteristics of lower digestible starch and higher amylose and dietary fiber content in chickpea compared to regular wheat starch support the blood sugar-improving effect of chickpeas [45]. Furthermore, their high-protein and resistant starch content has been reported to stimulate intestinal hormones such as GLP-1, GIP, and PYY [46,47,48]. Both GLP-1 and GIP stimulate insulin secretion, aiding in post-meal blood glucose concentration. 

Regarding the processing methods of chickpeas, many studies have revealed differences in efficacy between whole chickpeas and pureed or ground chickpeas [42]. This appears to be attributed to starch bio-accessibility based on cell wall integrity [42,49,50]. Actually, the extent of intracellular starch digestion from chickpeas is largely dependent on cell wall integrity, which acts as a barrier regulating hydration and controlling the permeability to α-amylase [38,49,50]. Consequently, the starch granules in intact chickpea cells are generally less susceptible to gelatinization and amylolysis, highlighting the underpinning mechanism of their lower post-prandial glucose response.

Additionally, in some studies, not only chickpeas but also lentils, navy beans, black beans, and yellow peas were compared [35,38,39,41]. As a result, the second-meal effect of chickpeas could be observed. Only chickpeas and lentils showed a blood glucose-lowering effect in the second meal after consumption, while other pulse crops did not exhibit such an effect. This suggests that the variation in the second-meal effects of pulses is not influenced by differences in the overall content of other macronutrients. In other words, the second-meal effect of chickpeas is not attributed to the post-consumption blood glucose response or their low glycemic index characterization.

What is unique is that many included studies have also revealed results related to appetite. In fact, several studies have shown that chickpea-based meals actually reduce appetite rates. In some studies, research on hormones related to appetite, such as GLP-1, leptin, and ghrelin, has been conducted, and the results have mostly indicated a positive impact [46,47,48]. Especially, intestinal hormones such as PYY are known to increase the feeling of fullness [47]. Furthermore, according to the glucostatic theory, it is also known to regulate food intake through the hypothalamic mechanism that triggers satiety when blood glucose levels increase [28]. However, there is variation across studies in these findings, and since meta-analysis has not been conducted, further research is needed in the future.

Based on these results, we can contemplate the incorporation of a diabetic diet utilizing chickpeas. In fact, according to many guidelines, diets for diabetic patients recommend avoiding processed foods, refined grains, processed red meats, and sugar-sweetened drinks [51,52,53]. Instead, they advocate for the consumption of fiber, vegetables, and yogurt [51,52,53]. Especially for individuals with diabetes, it is advised to steer clear of refined carbohydrates in order to enhance the quality of nutrients consumed [51,54]. From this perspective, incorporating whole chickpeas into the diet serves as a method to reduce refined carbohydrate intake by consuming whole grains and improving the quality of nutrients. Simultaneously, it offers a way to deliver less glucose to the circulatory system. Furthermore, the appetite-related benefits of chickpeas related to hormones could potentially have a positive impact on weight loss and appetite control for individuals with diabetes.

However, this study has certain limitations. Due to the nature of the research, maintaining consistency in the control group is not feasible. Particularly, as it involves dietary interventions, achieving uniformity in nutritional components is challenging, potentially compromising the intricacy of the study.

There was a limited number of studies included in the meta-analysis. Due to the small sample size, conducting quantitative analyses, such as Egger’s or Begg’s tests for publication bias, was deemed unfeasible and constrained.

## 5. Conclusions

A systematic review of 11 studies revealed that chickpea consumption could lead to improved insulin iAUC. Additionally, three of the four studies revealed that chickpeas could improve insulin iAUC.

However, the effect of insulin iAUC of chickpeas compared to wheat was not significant. Due to the limited number of studies included in the meta-analysis, caution is required in interpretation. Notably, many studies have focused on appetite, possibly attributed to hypothalamic mechanisms related to blood glucose changes and the influence of hormones such as GLP-1, GIP, PYY, ghrelin, and leptin. However, as of now, since a meta-analysis has not been conducted, further additional research is needed for a clearer confirmation of the effects.

Based on these characteristics, we can propose a diet that can help control blood sugar. In fact, for diabetes patients, there is often a need for blood sugar and appetite regulation. Utilizing the effects highlighted in this study regarding chickpeas could potentially contribute to their health management.

## Figures and Tables

**Table 1:** Nutritional ingredients of control groups and intervention groups.

Study ID	Nutritional Ingredients	Intervention	Control	
Nestel P (2004) [34]	Energy (kJ/day)	7424 ± 2938	7524 ± 3947	
	Total fat (g/day)	62 ± 34	67 ± 44	
	Saturated fat (g/day)	22 ± 14	26 ± 19	
	Monounsaturated fat (g/day)	20 ± 13	24 ± 17	
	Polyunsaturated fat (g/day)	11 ± 7	10 ± 7	
	Protein (g/day)	83 ± 32	89 ± 49	
	Carbohydrates (g/day)	222 ± 81	211 ± 100	
	Fiber (g/day)	33 ± 8	26 ± 13	
	Cholesterol (mg/day)	200 ± 119	235 ± 167	
Johnson SK (2005) [37]	Energy (kJ)	1342	1251	
	Protein (g)	11	9	
	Fat (g)	7	6	
	Available carbohydrates (g)	50	50	
	Total dietary fiber (g)	6	3	
Mollard RC (1) (2011) [38]	Energy (kcal)	603.2	604.1	
	Weight (g)	757.7	446.5	
	Energy density (kcal·g^−1^)	0.8	1.4	
	Volume (mL)	850.0	650.0	
	Available carbohydrates (g/100 g)	98.7	100.4	
	Fiber (g)	18.3	2.8	
	Protein (g)	26.1	22.8	
	Fat (g)	12.5	12.6	
Mollard RC (2) (2012) [39]	Energy (kJ/100 g)	324.5	322.0	
	Fat (g/100 g)	1.7	1.3	
	Available carbohydrates (g/100 g)	12.0	13.7	
	Fiber (g/100 g)	2.1	0.9	
	Protein (g/100 g)	3.5	2.6	
Mollard RC (3) (2014) [35]	Energy (kcal)	300	300	
	Available carbohydrates (g)	47.8	64.0	
	Protein (g)	16.1	9.9	
	Fiber (g)	11.3	2.8	
	Fat (g)	4.9	0.5	
Winham DM (2017) [36]	Energy (kcal)	258.0	232.0	
	Carbohydrates (g)	53.1	49.5	
	Available CHO (g)	47.6	49.5	
	Fiber (g)	5.5	0.7	
	Protein (g)	9.2	4.8	
	Fat (g)	2.3	0.5	
Zurbau A (2019) [40]			Potato	Pasta
	GI	28	85	45
	GL	14	42	42
	Energy (kcal)	418	423	462
	Available CHO (g)	50	50	94
	Dietary fiber (g)	20	6	5
	Fat (g)	14	15	2
	Protein (g)	23	22	17
	Na (g)	1.18	1.18	1.18
Alie J. Johnston (2020) [41]	Energy (kcal)	202.0	200.0	
	Available CHO (%db)	77.2	94.7	
	Protein (%db)	12.7	5.2	
	Resistant starch (%db)	0.2	0.2	
	Insoluble dietary fiber (%db)	5.5	2.3	
	Soluble dietary fiber (%db)	2.0	0.6	
	Starch damage (%db)	61.8	73.3	
	Fat (%db)	3.4	1.4	
Hafiz MS (2022) [42]	Weight (g)	250.0	425.0	
	CHO (g)	50.0	50.0	
	Fiber (g)	15.3	4.7	
	Fat (g)	8.0	8.0	
	Protein (g)	19.3	6.2	
	Salt (g)	0.8	0.8	
	Energy (kJ)	1447.6	1241.9	
Bajka BH (2023) [43]	Energy (kJ/serving)	1823.1	1301.8	
	Protein (g/serving)	27.16	12.88	
	Fat (g/serving)	8.84	3.33	
	Starch (g/serving)	42.5	45.3	
	Available CHO (g/serving)	48.2	48.1	
	Available dietary fiber (g/serving)	10.65	2.64	
	Available sodium (g/serving)	0.752	0.419	

**Table 2:** Details of included studies.

Study ID	Nation	Study Design	Period	Sample Size (F/M)	Experimental Group	Control Group	Outcome Measurement	Assessment
Nestel P (2004) [34]	Australia	Crossover RCT	6 weeks	19 (12/7) middle-aged people	Chickpea-based diet	Wheat-based diet	Blood glucose	Positive
Johnson SK (2005) [37]	Australia	Crossover RCT	1 week	11 (2/9) healthy people	Chickpea flour bread	Wheat bread (White bread)	(1) IAUC glucose (2) IAUC insulin	Positive
Mollard RC (1) (2011) [38]	Canada	Crossover RCT	5 weeks	25 (0/25) healthy males	Chickpeas *	Pasta and sauce *	Blood glucose iAUC	Positive
Mollard RC (2) (2012) [39]	Canada	Crossover RCT	4 weeks	24 (0/25) healthy males	Macaroni and chickpeas *	Macaroni and cheese *	Blood glucose iAUC	Positive
Mollard RC (3) (2014) [35]	Canada	Crossover RCT	5 weeks	15 (0/15) healthy males	Chickpeas *	Wheat bread * (White bread)	Blood glucose iAUC	Positive
Winham DM (2017) [36]	USA	Crossover RCT	3 weeks	12 (12/0) healthy females	Chickpeas and white rice	Rice only	(1) Glucose iAUC (2) Insulin iAUC	Positive: (1) NSD: (2)
Zurbau A (2019) [40]	Canada	Crossover RCT	Over 12 days	17 (9/8) healthy adults	Chickpeas **	Potato **	Blood glucose iAUC	Positive
						Pasta	Blood glucose iAUC	Positive
Alie J. Johnston (2020) [41]	Canada	Crossover RCT	5 days	26 (12/14) normoglycemic adults	Chickpeas	Corn	Plasma glucose iAUC Insulin iAUC	Positive
Hafiz MS (2022) [42]	UK	Crossover RCT	2 weeks	13 (9/4) normoglycemic adults	Whole chickpeas	Mashed potatoes	Blood glucose iAUC	Positive
Bajka BH (2023) [43]	UK	Crossover RCT	Over 12 days	20 (10/10) healthyindividuals	60% CCP and 40% wheat flour ***	100% wheat flour ***	(1) Plasma glucose iAUC (2) Insulin iAUC	Positive

iAUC: integrated area under the curve; CCP: cellular chickpea powder; NSD: not significantly different; * homogenized with food processor. ** mashed and prepared with water. *** no significant differences in the participant sensory scores in taste or texture.

## Data Availability

The data presented in this study are available on request from the corresponding authors (Y.O. and A.K.).

## Appendix A

**Table A1:** Search strategy in Cochrane library.

NO.	Search Strategy	Item
#1	Cicer arietinum OR chickpea OR chick pea	119
#2	MeSH descriptor: [Diabetes Mellitus] explode all trees	45,956
#3	MeSH descriptor: [Blood Glucose] explode all trees	19,279
#4	(“Randomized Controlled Trial”):pt OR (“Controlled Clinical Trial”):pt OR (randomized):ti,ab,kw OR (placebo):ti,ab,kw OR (randomly):ti,ab,kw OR (trial):ti,ab,kw	140,645
#5	#1 AND (#2 OR #3) AND #4	19

**Table A2:** Search strategy in PubMed.

NO.	Search Strategy	Item
#1	Cicer arietinum OR chickpea OR chick pea	3547
#2	Diabetes mellitus[Mesh] OR blood sugar[Mesh]	605,155
#3	Randomized Controlled Trial OR Controlled Clinical Trial OR randomized OR placebo OR randomly OR trial	2,784,527
#4	#1 AND #2 AND #3	35

**Table A3:** Search strategy in Embase.

NO.	Search Strategy	Item
#1	. ‘cicer arietinum’/exp OR ‘cicer arietinum’ OR ‘chickpea’/exp OR chickpea OR ‘chick pea’	3661
#2	. ‘diabetes mellitus’ OR diabetes OR ‘blood sugar’ OR ‘blood glucose’	1,561,079
#3	. ‘randomized controlled trial’/exp AND ‘randomized controlled trial’:it OR ‘controlled clinical trial’:it OR randomized:ti,ab,kw OR placebo:ti,ab,kw OR randomly:ti,ab,kw OR trial:ab,ti OR ‘clinical trials’:ti,ab,kw	2,442,971
#4	. #1 AND #2 AND #3	43

**Table A4:** Search strategy in KoreaMed.

NO.	Search Strategy	Item
#1	Search ( (“cicer arietinum”[ALL]) OR (“chickpea”[ALL]) OR (“chick pea”[ALL]))	3
#2	Search ( (diabetes mellitus[MH]) OR (blood sugar[MH]) OR (“diabetes”[ALL]))	11,533
#3	Search ( (“randomized”[ALL]) OR (“clinical”[ALL]) OR (“controlled”[ALL]) OR (“trials”[ALL]))	1100
#4	#1 AND #2 AND #3	0

**Table A5:** Search strategy in RISS.

NO.	Search Strategy	Item
#1	cicer arietinum|chickpea|chick pea|병아리콩	920
#2	blood sugar|blood glucose|diabetes|diabetes mellitus|혈당|당뇨	110,535
#3	#1 AND #2	20

병아리콩: chickpea, 혈당: blood sugar, 당뇨: diabetes.

**Table A6:** Search strategy in KISS.

NO.	Search Strategy	Item
#1	“전체 = ““cicer arietinum”“ or 전체 = ““chickpea”“ or 전체 = ““병아리콩”“	38

전체: total, 병아리콩: chickpea.

**Table A7:** Search strategy in ScienceOn.

NO.	Search Strategy	Item
#1	“cicer arietinum”|”chickpea”|”chick pea”|”병아리콩”	13,510
#2	“blood sugar”|”blood glucose”|”diabetes”|”diabetes mellitus”|”혈당”|”당뇨”	1,024,818
#3	“Randomized Trials”|”Controlled trials”|”Randomized Clinical Trials”|무작위 임상|무작위 시험|placebo|Randomized	332,557
#4	#1 AND #2 AND #3	2

병아리콩: chickpea, 혈당: blood sugar, 당뇨: diabetes, 무작위 임상: randomized trials, 무작위 시험: randomized trials.
